# Management of a Nonvital Young Permanent Tooth by Pulp Revascularization

**DOI:** 10.5005/jp-journals-10005-1268

**Published:** 2015-02-09

**Authors:** Vidya Chandran, Varghese Chacko, G Sivadas

**Affiliations:** Former Postgraduate Student, Department of Pedodontics and Preventive Dentistry Manipal College of Dental Sciences, Manipal University Mangalore, Karnataka, India; Associate Professor, Department of Pedodontics and Preventive Dentistry Manipal College of Dental Sciences, Manipal University Mangalore, Karnataka, India; Senior Lecturer, Department of Pedodontics and Preventive Dentistry, Sree Mookambika Institute of Dental Sciences, Kanyakumari, Tamil Nadu, India

**Keywords:** Pulp revascularization, Young permanent teeth open apex, Nonvital.

## Abstract

This report presents the case of a 10-year-old patient with a nonvital young permanent tooth which was managed by pulp revascularization. Following disinfection of the canal by irrigation with NaOCl and use of a triantibiotic paste, a scaffold was created by inducing the formation of a blood clot within the canal. At the subsequent follow-up visits, the patient was asymptomatic, with normal response to percussion, normal periodontal probing depths, and no abnormal mobility. The radiographs showed evidence of continued apical root development with increase in root length, signs of apical closure and increase in thickness of dentinal walls. Thus, this case adds to the growing evidence supporting the revascularization approach as an option for management of nonvital young permanent teeth.

**How to cite this article:** Chandran V, Chacko V, Sivadas G. Management of a Nonvital Young Permanent Tooth by Pulp Revascularization. Int J Clin Pediatr Dent 2014;7(3):213-216.

## INTRODUCTION

Management of nonvital young permanent teeth present the clinician with numerous restorative and endodontic challenges. Traditionally, the most popular method for managing such teeth has been by apexification using calcium hydroxide. More recently, the use of MTA to create an apical barrier followed by placement of a bonded core within the canal to strengthen the weakened roots has been the standard of care for nonvital teeth with open apices.^1^ Even though both these approaches have been used successfully over the years, both modalities have few important well documented drawbacks, one of which is the lack of continued root development.

This has led clinicians and researchers to search for alternate strategies to promote continued root development even in young permanent teeth which have lost pulp vitality. One such strategy is the pulp revascularization approach. Early attempts yielded inconsistent results and histologic examinations failed to demonstrate the regeneration of pulp-dentin complex.^2^ Recent improvements in dental materials and better understanding of the regenerative potential of pulp has led to a resurgence of interest in pulp regeneration techniques^3^ with various groups publishing case reports describing regenerative endodontic procedures carried out on nonvital young permanent teeth. The promising outcome from these reports has caused a paradigm shift in the approach to management of immature teeth. Now there is greater emphasis on conservative, biologically based methods that allow regeneration of a functional pulp-dentin complex.

This report presents the case of a nonvital young permanent central incisor in a 10-year-old boy which was treated by revascularization. The case report hopes to add to the body of growing evidence supporting the revascularization approach as an option for management of nonvital young permanent teeth.

## CASE REPORT

**Fig. 1:**
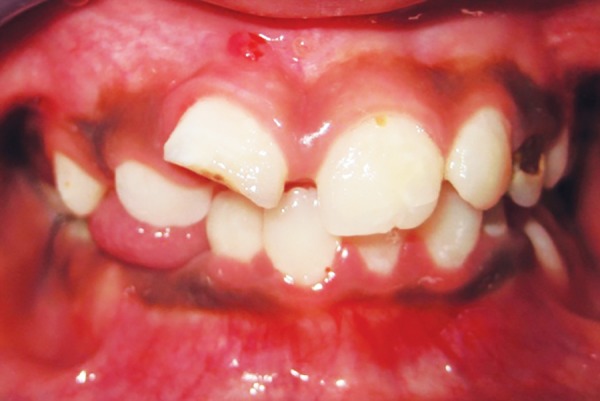
Complicated crown fracture with sinus tract in relation to upper right central incisor

**Fig. 2:**
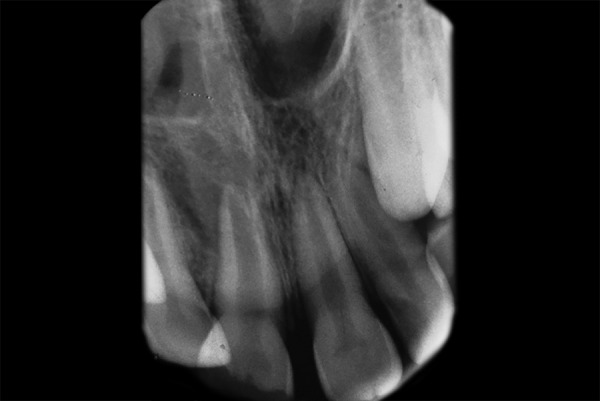
Preoperative IOPA showing open apex and thin dentinal walls

A 10-year-old male reported with a complaint of fractured upper front tooth associated with occasional mild, spontaneous pain for the past 4 to 6 months. On clinical examination, the right upper central incisor presented with a complicated crown fracture and was tender on percussion with no abnormal mobility. A draining sinus tract was observed in the buccal mucosa in relation to the upper right central incisor (Fig. 1). The tooth did not respond to cold test or electric pulp test (EPT). Radiographic examination revealed the tooth to have an incomplete root with an open apex with no signs of any periapical changes (Fig. 2). The patient's history revealed that he had fractured the tooth following a fall at home 14 months back and had not sought treatment for the same until now.

Taking into consideration the incomplete root development and nonvital status of the pulp it was decided to attempt pulp revascularization. Before proceeding with the treatment the risks and possible outcomes of the treatment were comprehensively discussed with the parent and consent was obtained.

**Fig. 3:**
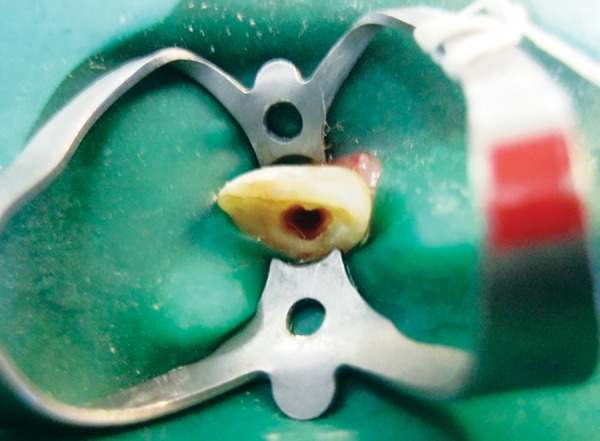
Blood clot formation

**Fig. 4:**
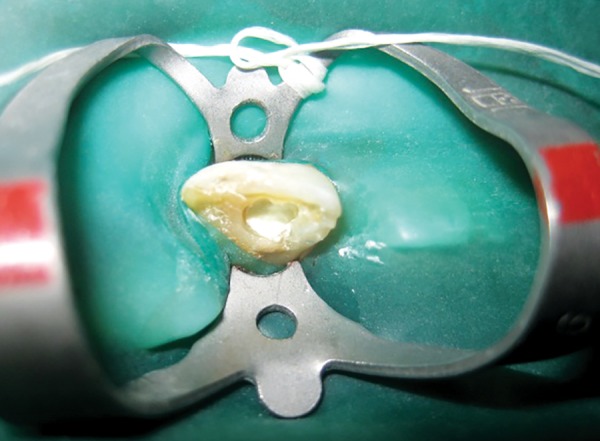
Placement of MTA

We followed the technique as described by Banchs and Trope.^4^ Following administration of local anesthesia (2% lignocaine with adrenalin, 1:200000) and isolation with rubber dam, access was obtained to the pulp space. The canal was not instrumented, but was disinfected by copious irrigation with 5.25% sodium hypochlorite, dried with sterile paper points and flled with a creamy paste of metronidazole, ciprofoxacin and minocycline mixed in a ratio of 1:1:1 in propylene glycol. The access cavity was then sealed with resin modified GIC. When the patient returned after 4 weeks, the sinus tract had healed. After administration of local anesthesia (2% lignocaine with adrenalin, 1:200000), the antibiotic paste was removed by irrigation with saline and 5.25% hypochlorite. Bleeding was induced into the canal by stimulation of tissue beyond the apex using a sterile endodontic file. After waiting for approximately 15 minutes, to allow the blood clot to reach a level approximating the cementoenamel junction (Fig. 3), white MTA (Angelus) was mixed and placed over the clot (Fig. 4). A moist cotton pellet was placed over the MTA and access was sealed with Resin modified glass ionomer. After 2, days the cotton pellet was removed and the tooth was restored with GIC and composite resin.

**Fig. 5:**
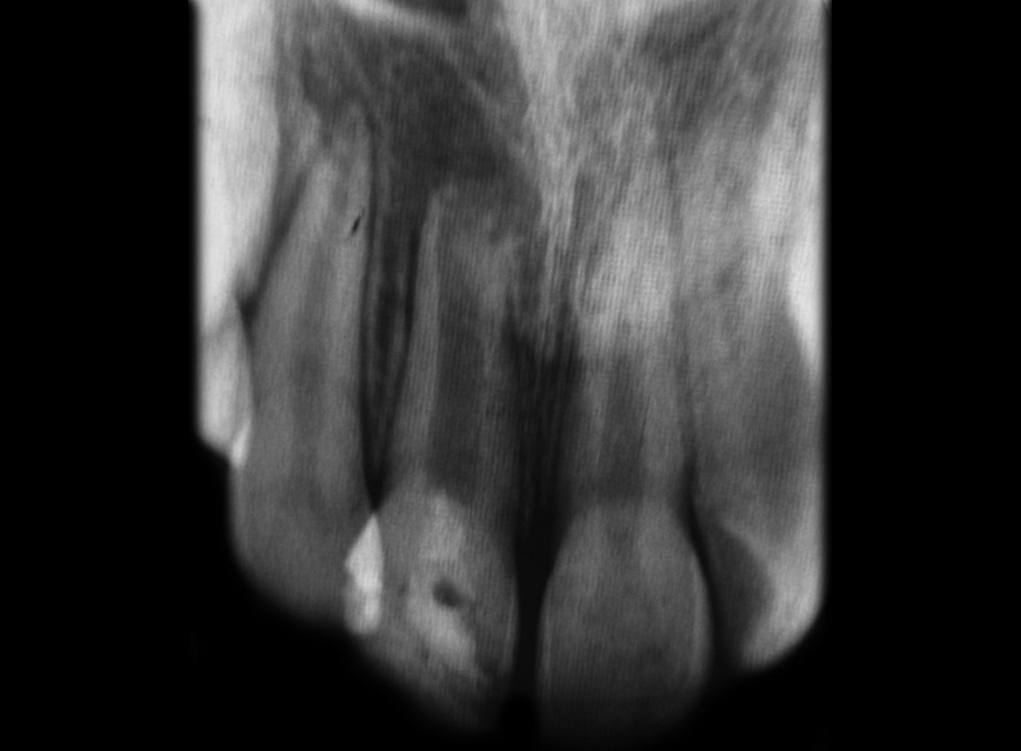
IOPA at 6 months showing signs of continued root development

**Fig. 6:**
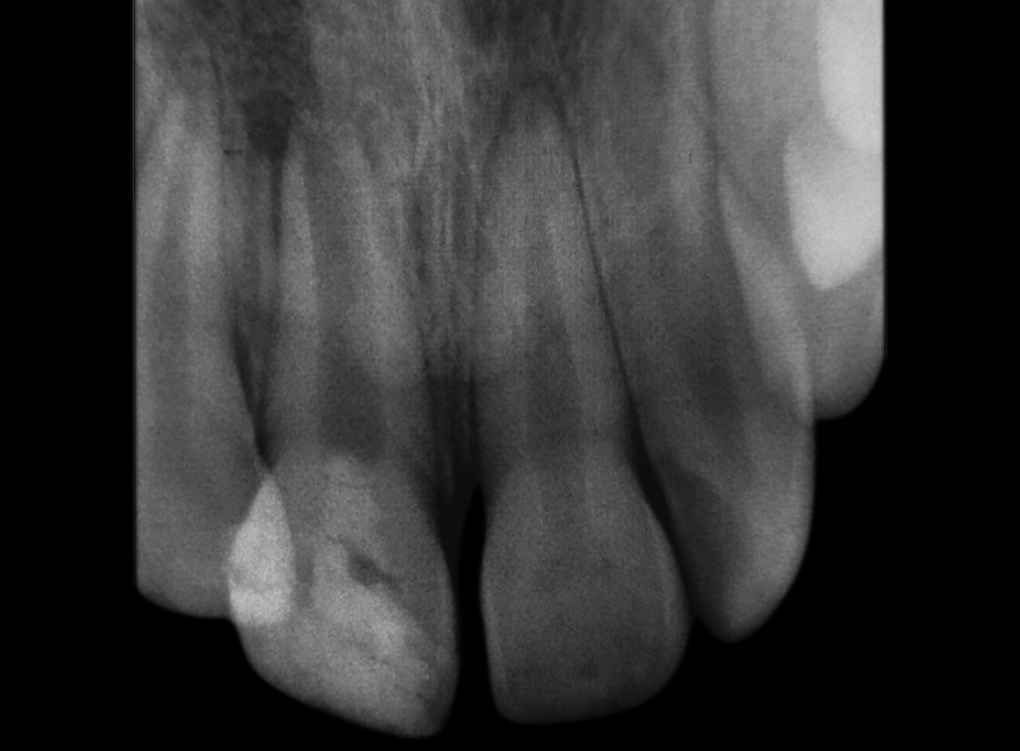
IOPA at 1 year showing evidence of increase in root length, increased dentinal wall thickness and signs of apical root development

The patient was asked to report for follow-up evaluations at 3 months intervals. Clinically the tooth remained asymptomatic, with normal response to percussion, normal periodontal probing depths, and no abnormal mobility at the end of 3, 6 and 12 months. However, the tooth did not respond to cold test or EPT. Radiographic examination revealed normal peri-radicular structures with signs of increased root length and root wall thickening (Figs 5 and 6).

## DISCUSSION

Investigators have identified a unique set of circumstances that exist following the replantation of an avulsed tooth which enable revascularization.^5^ Firstly, the young teeth usually have a wide open apex and short root which favor migration of cells from the periapical region into the pulp space. Secondly, the pulp even though necrotic, is usually not infected and this acts as a scaffold into which tissue can grow. Finally, the crown is usually intact thus preventing the invasion of bacteria into the pulp space.

Various case reports have reported the successful revascularization of pulp tissue in infected nonvital young permanent teeth after replicating conditions similar to those found in avulsed young permanent teeth.^4,6-8^ In the present case we obtained disinfection of the canals by copious irrigation with 5.25% sodium hypochlorite and use of a triantibiotic paste which was described by Hoshino et al.^9^ The various other irrigants which have been used successfully for disinfection include 1.25% sodium hypochlorite and povidone iodine,^10^ chlorhex-idine^10^ and 5% sodium hypochlorite with 3% hydrogen peroxide.^11^ While many reports have successfully used the triple antibiotic preparation similar to the one used in our case, concerns of tooth discoloration caused by the minocycline^12^ component of the paste has led to the use of various modifications of this mixture. Replacement of minocycline with cefaclor 13 or amoxicillin^14^ and use of a bi-antibiotic paste free of minocycline^13^ have proven to be effective for disinfection. Also, some reports have successfully used Ca(OH)_2_ for disinfection.^6^ However, placement of the calcium hydroxide only in the coronal half of the root canal is critical for a successful outcome.^15^ This might be because of the cytotoxic effects of calcium hydroxide on the stem cells if placed in the apical region of the canal.

We provided a scaffold for the ingrowth of tissue by inducing bleeding into the canal space and allowing for the formation of a clot at approximately the level of the cementoenamel junction. Bleeding was induced by stimulation of the periapical tissue with an appropriately sized file under local anesthesia. Inducing bleeding and formation of the blood clot at the right level can often be difficult.^16^ Reports have documented that more predictable bleeding can be induced by using a local anesthetic without vasoconstrictor.^17^ Another method which has been suggested to be useful in inducing bleeding is to use a slightly bent file dipped in a calcium chelator like ethylenediaminetetraacetic acid.^17^ The search for more predictable scaffolds have led to the successful use of platelet rich plasma (PRP) 18 and platelet rich fibrin (PRF) 19 in regenerative endodontic procedures.

A bacteria tight seal was obtained in our case by use of MTA over the clot and sealing the access with GIC and a bonded composite restoration. Limiting the apical extent of the MTA plug can prove to be technically challenging.^16^ Collapse of MTA into the canal due to lack of strength of the clot can adversely affect the outcome of the case.^17^ A technique that has proven to be useful in controlling the placement of MTA is the placement of a collagen matrix over the clot prior to condensation of MTA.^17^

At present, there is still considerable uncertainty as to the nature of tissue that reoccupies the pulp space.^5^ Torabinejad and Faras reported the regeneration of pulp like tissue in one of their cases where they used PRP as a scaffold.^18^ However, most histologic studies done on animal models, suggest that the tissue which reoccupies the pulp space is mostly periodontal in nature.^19^ The outcome of this procedure seems to be tissue repair rather than tissue regeneration.

At the end of 1 year, even though the tooth did not respond to cold test or electric pulp test, there were definite signs of apical root development with narrowing of the canal space and thickening of lateral walls at the apical one-third. These findings are consistent with the reports of Shah N et al^8^ and Thibodeau and Trope.^13^ Banchs and Trope^4^ in another case, reported positive response to the cold test at the 2 years follow-up period. Unfortunately, our patient did not report for follow-up after 1 year, despite stressing the importance of long-term follow-up in the success of this treatment. Compliance with appointments is a significant problem when dealing with patient populations visiting dental schools and this is a significant factor that should be considered in case selection for this procedure.^16^

Even though many of the steps in the revasculari-zation procedure are technically challenging and the evidence supporting the successful outcome of this procedure is mainly from case reports, this is a procedure with huge potential and benefits for the patient. Hence, this is a technique that definitely merits a try when one has to treat nonvital young permanent teeth, as even if the treatment fails, the operator can still resort to the traditional methods for management of these cases.
